# A machine learning pipeline revealing heterogeneous responses to drug perturbations on vascular smooth muscle cell spheroid morphology and formation

**DOI:** 10.1038/s41598-021-02683-4

**Published:** 2021-12-02

**Authors:** Kalyanaraman Vaidyanathan, Chuangqi Wang, Amanda Krajnik, Yudong Yu, Moses Choi, Bolun Lin, Junbong Jang, Su-Jin Heo, John Kolega, Kwonmoo Lee, Yongho Bae

**Affiliations:** 1 Department of Pathology and Anatomical Sciences, Jacobs School of Medicine and Biomedical Sciences, University at Buffalo, State University of New York, Buffalo, NY 14203 USA; 2 Department of Biomedical Engineering, Worcester Polytechnic Institute, Worcester, MA 01609 USA; 3 Department of Computer Science, Worcester Polytechnic Institute, Worcester, MA 01609 USA; 4 Vascular Biology Program, Boston Children’s Hospital, Boston, MA 02115 USA; 5 Department of Orthopedic Surgery, Perelman School of Medicine, University of Pennsylvania, Philadelphia, PA 19104 USA; 6 Department of Surgery, Harvard Medical School, Boston, MA 02115 USA

**Keywords:** Cell biology, Cell adhesion, Cell growth, Cell signalling, Cellular imaging, Mechanisms of disease, Computational biology and bioinformatics, Computational models, Functional clustering, Image processing

## Abstract

Machine learning approaches have shown great promise in biology and medicine discovering hidden information to further understand complex biological and pathological processes. In this study, we developed a deep learning-based machine learning algorithm to meaningfully process image data and facilitate studies in vascular biology and pathology. Vascular injury and atherosclerosis are characterized by neointima formation caused by the aberrant accumulation and proliferation of vascular smooth muscle cells (VSMCs) within the vessel wall. Understanding how to control VSMC behaviors would promote the development of therapeutic targets to treat vascular diseases. However, the response to drug treatments among VSMCs with the same diseased vascular condition is often heterogeneous. Here, to identify the heterogeneous responses of drug treatments, we created an in vitro experimental model system using VSMC spheroids and developed a machine learning-based computational method called HETEROID (heterogeneous spheroid). First, we established a VSMC spheroid model that mimics neointima-like formation and the structure of arteries. Then, to identify the morphological subpopulations of drug-treated VSMC spheroids, we used a machine learning framework that combines deep learning-based spheroid segmentation and morphological clustering analysis. Our machine learning approach successfully showed that FAK, Rac, Rho, and Cdc42 inhibitors differentially affect spheroid morphology, suggesting that multiple drug responses of VSMC spheroid formation exist. Overall, our HETEROID pipeline enables detailed quantitative drug characterization of morphological changes in neointima formation, that occurs in vivo, by single-spheroid analysis.

## Introduction

**Figure 1:**
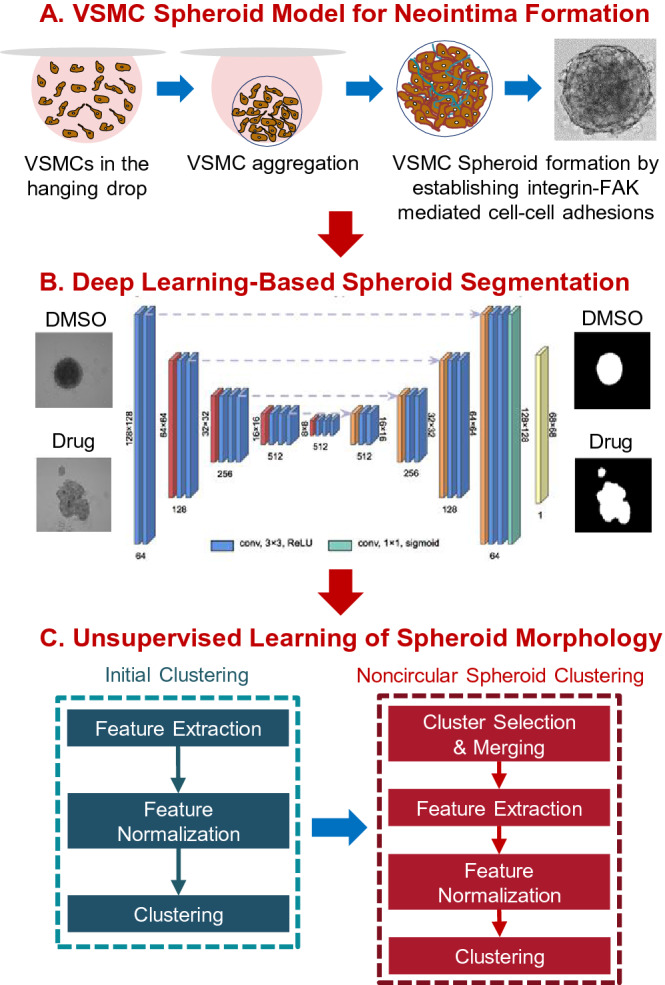
Overview of the HETEROID framework. VSMC spheroid images (**A**) were segmented by VGG16-U-Net semantic segmentation (**B**), and feature vectors were extracted for additional clustering analyses. A two-level clustering framework (**C**) was used to analyze the presence of morphological subpopulations of VSMC spheroids. Initial clustering analysis was focused on the roundness of the spheroids. Subsequent clustering analysis was focused on disrupted spheroids.

Vascular smooth muscle cells (VSMCs) play key roles in developmental, physiological, and pathological processes in the vessel wall. VSMC accumulation and proliferation occur during normal vascular development and repair of vascular injury^1–3^. However, pathologies arise when VSMCs continue to proliferate even after vascular remodeling has been completed. Uncontrolled VSMC accumulation and proliferation, which are the main pathologic features of vascular injury, restenosis, and atherosclerosis contribute to intimal thickening or neointima formation^1,4–12^. Thus, understanding how to control VSMC behaviors would advance the effort to treat vascular diseases. However, clinically effective drug targets for the prevention and treatment of restenosis and neointima formation have not been well established. Moreover, drug responses are frequently different among patients with the same vascular condition^13–21^. This phenomenon can be attributed to the heterogeneity of the underlying VSMC biology. In this paper, we present an experimental and computational pipeline called HETEROID (heterogeneous spheroid), which combines a VSMC spheroid model (Fig. 1A) and machine learning (ML) analysis (Fig. 1B,C) to deconvolve the heterogeneous drug responses on VSMC behaviors involved in neointima formation.

VSMC interactions among cells (cell-cell adhesion) and between cells and the surrounding extracellular matrix (ECM; cell-ECM adhesion) contribute to neointima formation^10,11^. Integrin-mediated FAK phosphorylation^10,22^ facilitates cell-cell or cell-ECM adhesion as well as cell proliferation during VSMC-mediated neointimal hyperplasia and formation^11,12,23–25^. FAK controls these cellular processes by activating its downstream small GTPases Rac, Rho, and Cdc42. First, the activation of Rac by FAK promotes cell-cell adhesion and cell proliferation^11,12,26^. Moreover, a previous in vivo study found that VSMC-specific deletion of Rac reduced cell proliferation and neointima formation^11,12^. Second, Rho activity has been shown to not only promote cell contraction and the formation of stress fibers and focal adhesion but also control VSMC proliferation^27–30^. A previous study showed that Rho inhibition increased neointima formation and prevented restenosis by increasing VSMC apoptosis after vascular injury^31^. Last, FAK also promotes the activation of Cdc42^32–34^. Li et al. showed that the inactivation of Cdc42 disrupted cell-cell adhesion and cell proliferation in cardiomyocytes^34^. Collectively, these studies suggest that FAK, Rac, Rho, and Cdc42 are potentially involved in neointima formation; however, their differential regulation in VSMC-mediated neointima formation remains unexplored.

In this study, we created VSMC spheroids*,* which are spherical cellular aggregates consisting of multiple layers of VSMCs^35,36^, using a hanging drop culture technique^37,38^ (Fig. 1A). This new spheroid model can mimic VSMC-mediated neointima formation in the vessel wall, similar to animal models^8,12,39,40^ that simulate human pathologies such as restenosis and atherosclerosis. Using this system, we characterized the effects of the inhibition of FAK phosphorylation and Rac, Rho, and Cdc42 activation on spheroid formation and morphology. To analyze the image data, we developed an ML framework that combines high-performance deep learning-based spheroid segmentation (Fig. 1B) and morphological clustering analysis (Fig. 1C) to identify the effects of various drug treatments on spheroid morphology and formation. To extract the morphological parameters of the spheroids, it is necessary to employ automated image analyses of microscopy images^41–46^. Therefore, we first applied a deep learning approach for spheroid segmentation from phase-contrast images. Specifically, we combine two deep learning architectures: U-Net and ImageNet-pretrained VGG16 models^47–50^. This transfer learning approach has been widely used to reduce the size of the training set and minimize overfitting^51–54^. After segmentation, various morphological features were extracted, and unsupervised learning was applied to identify distinct subpopulations of VSMC spheroids. Interestingly, our clustering analysis identified the morphological variations and subpopulations in drug responses among VSMC spheroids. Thus, our HETEROID pipeline can be used to quantitatively assess the effects of various drugs on the VSMC spheroid model for better characterization of morphological changes in neointima formation that occur in vascular diseases.

## Results

### Differential VSMC spheroid formation responses to FAK, Rac, Rho, or Cdc42 inhibition

**Figure 2:**
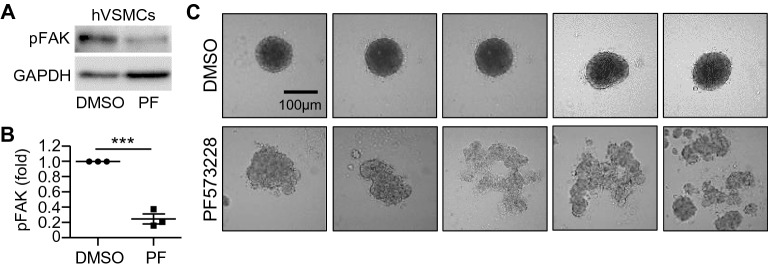
Inhibition of FAK phosphorylation differentially affects VSMC spheroid formation and morphology. Human VSMCs treated with FAK inhibitor (PF573228) or DMSO (vehicle control) in high-glucose DMEM containing 10% FBS were incubated for 24 h to generate human VSMC spheroids. Total cell lysates were immunoblotted (**A**) for phosphorylated FAK at Tyr397 (pFAK) and GAPDH. The bar graph displays the pFAK levels normalized to the DMSO control (**B**). (**C**) Cultures were imaged using an upright microscope. *n* = 3 (**A**–**B**) and *n* = 12 (**C**) biological replicates were used. ***p < 0.001.

Since FAK phosphorylation is critical in the regulation of both cell-cell adhesion and cell proliferation during the process of neointima formation^11,12,22,55,56^, we first tested the inhibition of FAK phosphorylation on VSMC spheroid formation by culturing human aortic VSMCs (Fig. 2) and mouse aortic VSMCs (Fig. S1) to form spheroids in the presence of either PF573228 (a selective FAK inhibitor)^11^ or DMSO (a vehicle control). After the spheroid images were acquired at 24 h, total cell lysates were prepared from VSMC spheroids. FAK phosphorylation at Tyr^397^ in both human VSMCs (Fig. 2A, B and Fig. S2A) and mouse VSMCs (Figs. S1A,B, S2B) was reduced by approximately 70–80% with 10 μM PF573228 treatment. We also confirmed that VSMC spheroid morphologies with DMSO are mostly spherical (Figs. 2C, S1C), as previously shown in other studies^35,36^. Inhibition of FAK phosphorylation significantly disrupted the spheroid formation of both human (Fig. 2C) and mouse VSMCs (Fig. S1C). Thus, FAK phosphorylation is required for normal VSMC spheroid formation. More interestingly, the PF573228-treated VSMC spheroids had widely varied morphologies in both human (Fig. 2C) and mouse VSMCs (Fig. S1C), indicating the existence of heterogeneity in the response to inhibition of FAK phosphorylation and suggesting the need for further image analysis using the ML approach (Fig. 1B,C).

**Figure 3:**
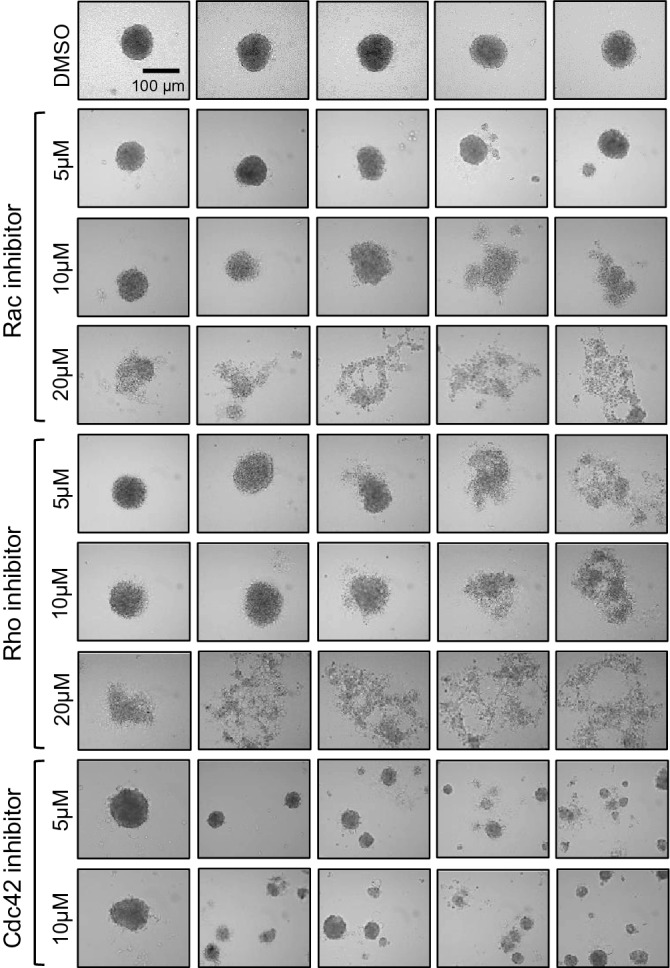
Inhibition of Rac, Rho, or Cdc42 activation differentially affects VSMC spheroid formation and morphology. Human VSMCs treated with Rac (EHT1864), Rho (Rhosin), and Cdc42 (ML141) inhibitors or DMSO (vehicle control) were used to generate human VSMC spheroids. VSMC spheroids were imaged after 24 h of incubation using an upright phase-contrast microscope.

The activities of Rac, Rho, and Cdc42 are essential for VSMC proliferation, vascular remodeling, and neointima formation^11,31,33,57^. Therefore, we tested the effects of the drug inhibition of Rac, Rho, and Cdc42 activation on VSMC spheroid formation using EHT1864 (a Rac inhibitor, 5–20 μM), Rhosin (a RhoA inhibitor, 5–20 μM), ML141 (a Cdc42 inhibitor, 5–10 μM), or DMSO. Note that this range of concentrations was previously used and validated^58–63^. The formation of VSMC spheroid was undisrupted or slightly disrupted with 5 or 10 μM EHT1864 (Fig. 3, 2nd and 3rd rows) and 5 or 10 μM Rhosin (Fig. 3, 5th and 6th rows), whereas spheroid formation was significantly disrupted with a higher dose (20 μM) of EHT1864 (Fig. 3, 4th row) or Rhosin (Fig. 3, 7th row) and 5–10 μM ML141 (Fig. 3, 8th and 9th rows), compared to DMSO-treated VSMC spheroids (Fig. 3, 1st row). Interestingly, the inhibition of Cdc42 activation resulted in the formation of multiple small cell aggregates, distinct from the disrupted morphologies in cultures treated with FAK, Rac, and Rho inhibitors. More interestingly, we also observed that Rac-, Rho-, and Cdc42-inhibited human VSMC spheroids displayed substantially heterogeneous morphologies in human VSMC spheroids from relatively unperturbed to clearly disrupted ones. Collectively, these observations suggested the existence of some distinctive characteristics (morphological heterogeneity) among VSMC spheroids in response to the same and different drug treatments.

### Image segmentation of VSMC spheroid images using deep learning

**Figure 4:**
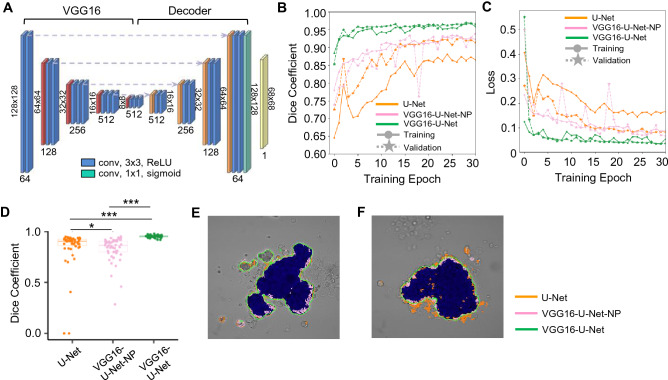
VGG16-U-Net-based image segmentation of VSMC spheroid morphology. (**A**) VGG16-U-Net structure. The training and validation curves of the Dice coefficient (**B**) and the loss function (**C**). (**D**) Comparison of the Dice coefficients among U-Net, VGG16-U-Net-NP, and VGG16-U-Net. *p = 0.015 and ***p < 10^–14^. The p-value was calculated by the Wilcoxon rank sum test. (**E**,**F**) The edges of the spheroid images were identified by U-Net, VGG16-U-Net-NP, and VGG16-U-Net. The blue regions represent the manually labeled ground-truth images.

To deconvolve the heterogeneity of morphological subpopulations of VSMC spheroids following drug treatments, we further examined spheroid morphological changes using ML-based image segmentation, followed by data clustering analysis to identify the presence of morphological subpopulations (clusters) resulting from the drug treatments. Our spheroid images were collected by a phase-contrast microscope, hence reducing the phototoxic effect of fluorescent probes and avoiding physical damage to the delicate samples due to handling and preparation. Therefore, conventional image segmentation algorithms based on intensity thresholding or edge detection are limited for robust edge detection, particularly for drug-treated spheroids. Thus, we used a deep learning structure, a U-Net integrated with the ImageNet- pretrained VGG16 model, called VGG16-U-Net^48^ (Fig. 4A), to train the deep neural network to segment the phase-contrast images of VSMC spheroids. First, spheroid boundaries were manually drawn on a subset of spheroid images using the Pixel Annotation Tool, and these annotated images were then used to train the VGG16-U-Net. When tested with new images outside the training set, the trained VGG16-U-Net identified spheroid boundaries with 96% accuracy at epoch 30, as demonstrated in the learning curves plotted as the Dice coefficient (Fig. 4B) and loss function using binary cross-entropy in training and validation datasets (Fig. 4C). The VGG16-U-Net produced a significantly higher segmentation accuracy than the standard U-Net (Fig. 4D). We also found that VGG16-U-Net significantly outperforms the non-pretrained version (VGG16-U-Net-NP) (Fig. 4D). This demonstrates the benefits of the model weights of VGG16 pretrained on ImageNet for the image segmentation (Fig. 4B–D). Visually, the trained VGG16-U-Net achieved an excellent segmentation performance in comparison to U-Net and VGG16-U-Net-NP (Fig. 4E,F).

### Clustering analysis reveals morphological subpopulations

**Figure 5:**
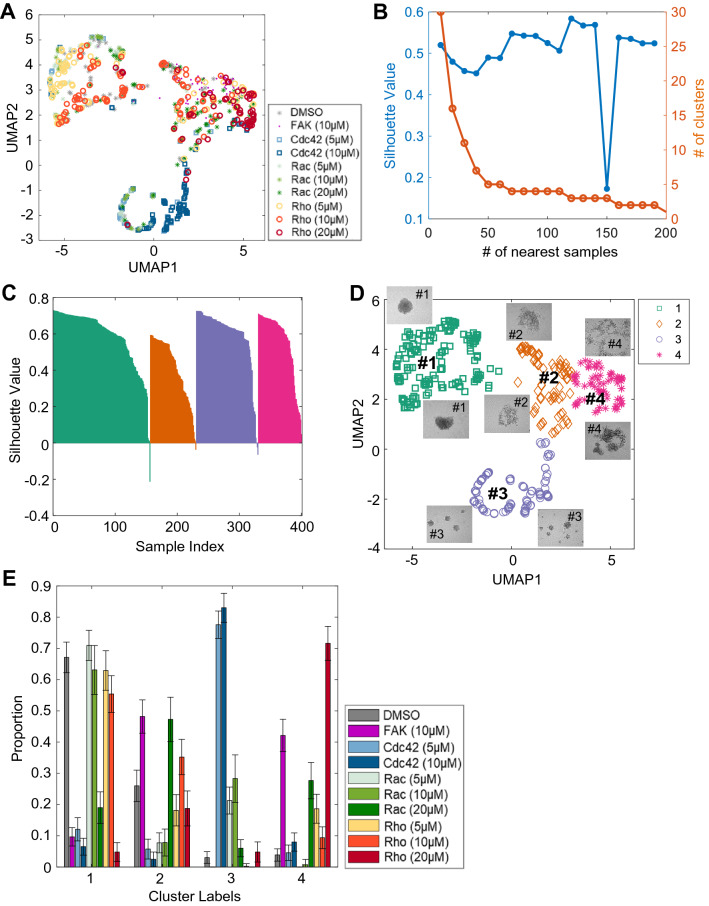
Initial morphological clustering analysis identifying distinct clusters in response to inhibitors of FAK, Rac, Rho, and Cdc42. (**A**) Four morphological features extracted from human VSMC spheroid images are displayed on a UMAP plot. The different colored markers are used to represent the various drug treatments: FAK (PF573228), Rac (EHT1864), Rho (Rhosin), and Cdc42 (ML141). (**B**) The silhouette values and the number of clusters on the training set were evaluated by varying the number of nearest samples. (**C**) Silhouette plots for the clustering results on the testing set. (**D**) Four morphological clusters plotted on the UMAP plot were observed based on the “roundness of the spheroid”. The different colored markers are used to represent the various morphological clusters with the drug treatments. The most representative images were overlayed on each cluster (2 images per cluster) in the UMAP plot. Clusters #1 and #3 showed spheroids with circular morphologies, and the spheroids in Clusters #2 and #4 showed noncircular and dispersed/disrupted morphologies. Only the samples in the testing set were used. (**E**) Average proportionality plot of the distribution of VSMC spheroids in each morphological cluster with the drug treatments from the repeated random splitting of training and testing sets. Only testing sets were used.

The morphological features of the spheroid images were extracted from the segmented binarized images without considering the grayscale information in the raw spheroid images. Five morphological features (the *eccentricity*, *solidity*, *extent*, *circularity*, and *number of colonies*; see “Methods” for details) were selected and displayed in a UMAP (uniform manifold approximation and projection) plot, a dimensionality reduction technique that visualizes a high-dimensional dataset by learning the data manifold^64^ (Fig. 5A). The data points scattered on the UMAP plot revealed the presence of morphological variations among the drug-treated VSMC spheroids.

The morphological feature vectors of the VSMC spheroids were further analyzed using a clustering analysis with the community detection algorithm^65^ to identify the distinct groups of spheroid images of similar morphological features. To determine the number of clusters, we first randomly split the data into training and testing sets in equal proportion. We varied the number of nearest samples, which is a hyperparameter in the community detection algorithm, to produce the clustering outcomes of different numbers of clusters in the training set. We calculated the quality of each clustering result by evaluating the silhouette value, which is a measure of how similar the morphology of a given spheroid is to its own cluster compared to other clusters. We chose four as the number of clusters based on the maximum silhouette values for the number of nearest samples (Fig. 5B). Then, we performed the same clustering analysis for the testing set using the same number of clusters. The silhouette plot of the final clustering analysis showed the least overlap between clusters and mostly positive silhouette values (Fig. 5C). The distribution of spheroid morphologies in each cluster is displayed on a UMAP plot (Fig. 5D), and the proportion of spheroids in each cluster for a given drug treatment is shown in Fig. 5E. Cluster #1 has stereotypical morphologies of unperturbed circular spheroids (Fig. 5D, inset). Clusters #2 and #4 are noncircular and dispersed or disrupted. In Cluster #3, the images showed small circular shapes but contained more than one spheroid.

Analysis of clustering with the training set gave similar results to the testing set (Fig. S3A,B). Furthermore, the robustness of the clustering results was tested by repeating the clustering 100 times using 100 different random splits of the training and testing datasets. We counted the cluster assignments of individual spheroid images from the repeated clustering, and assigned the majority cluster labels to them. Then we compared the majority cluster labels with the cluster labels of spheroid images in each clustering. As demonstrated in Fig. S3C, the cluster assignments were highly consistent (Cluster #1: 99.76%, Cluster #2: 97.24%, Cluster #3: 99.63%, Cluster #4: 96.57%). Also, clustering of the entire dataset without the data splitting exhibited similar results (Fig. S4).

### Different drug treatments produce spheroid subpopulations with different morphology profiles

For DMSO-treated controls, most (approximately 70%) were in Cluster (or Type) #1 (Fig. 5E), as were human VSMC spheroids treated with the lower inhibitor concentrations (5 or 10 µM Rac and Rho inhibitors, Fig. 5E). These results indicated that VSMC spheroids in this cluster maintained unperturbed circular morphology or had minimal effects with DMSO or lower concentrations of Rac and Rho inhibitors. In contrast, the proportion of human VSMC spheroids treated with 10 μM FAK inhibitor was reduced to 10% in Cluster #1 and increased up to approximately 50% Type 2 and 40% Type 4 (Fig. 5E). High-dose (20 μM) treatment with a Rac inhibitor produced spheroids that were 50% Type 2 and 25% Type 4. A high dose (20 μM) of a Rho inhibitor led to the production of spheroids that are 20% Type 2 and 70% Type 4, which are significantly fewer than and more than those for FAK and Rac inhibition, respectively. Interestingly, treatment with 5 and 10 μM Cdc42 inhibitor created spheroids that are approximately 80% Type 3. Although the spheroids in Type 3 showed circular morphologies, each culture contained multiple small spheroids. In the cultures in which FAK and Rac (20 μM) were inhibited, the spheroids were mostly Types 2 and 4 (noncircular and dispersed/disrupted, respectively), Rho-inhibited (20 μM) spheroids were mostly Type 4 (dispersed/disrupted), and Cdc42-inhibited spheroids were mainly Type 3 (small fragmented). Interestingly, however, the data shown in Fig. 5E also demonstrated that different morphologies of spheroids were generated within a single treatment (i.e., FAK inhibition created approximately 10% Type 1, 50% Type 2, 2% Type 3, and 40% Type 4 VSMC spheroids). Collectively, these results demonstrate that morphological subpopulations of spheroid formation respond differentially to drug treatments, suggesting different cellular states (i.e., proliferative and metabolic states) following drug treatment affecting the process of VSMC spheroid formation.

### Cluster analysis of noncircular spheroids reveals the differential effects of FAK, Rac, Rho, and Cdc42 inhibition

**Figure 6:**
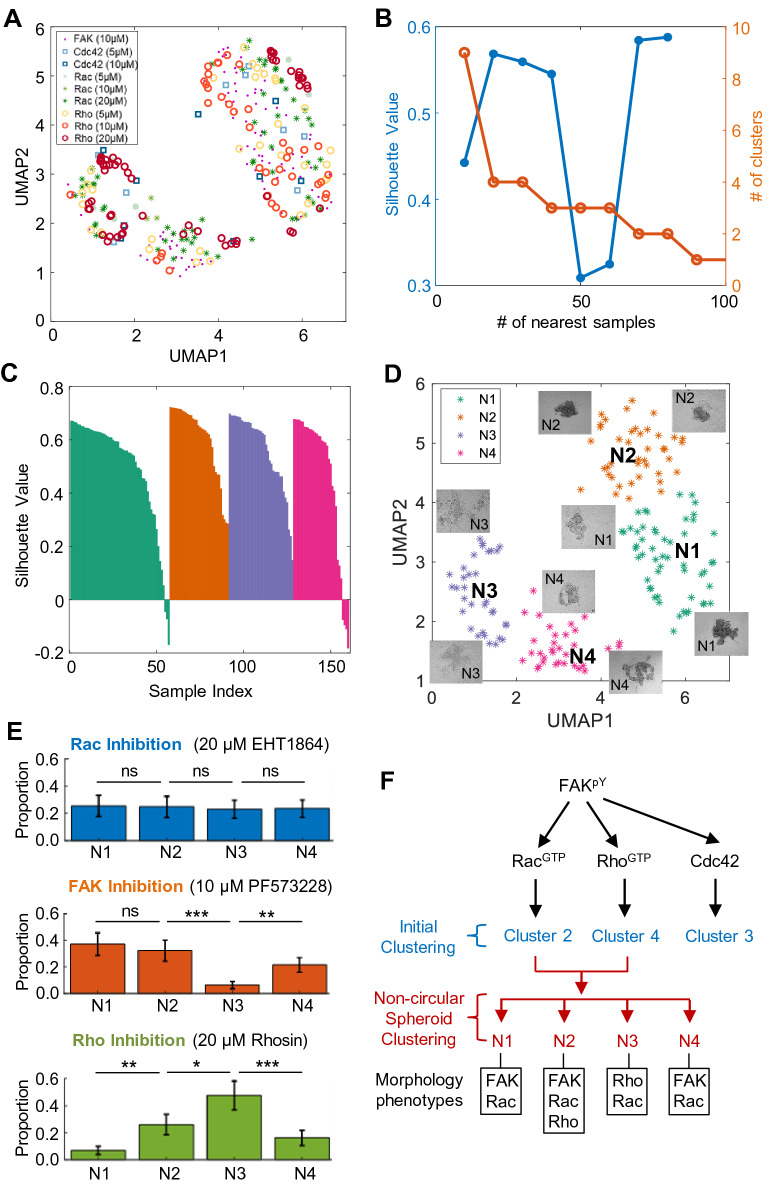
Clustering analysis of noncircular spheroids and the presence of distinct clusters with disrupted morphologies. (**A**) Fifteen morphological features extracted from human VSMC spheroid images from Clusters #2 and #4 were visualized on UMAP plots. The different colored markers are used to represent the various drug treatments. (**B**) The number of nearest samples between 10 and 100 was evaluated on the training set, and the silhouette values were plotted. A cluster number of 4 was selected. (**C**) The silhouette values and the number of clusters on the training set were evaluated by varying the number of nearest samples. (**D**) Four morphological clusters were observed in the testing set based on the noncircular spheroids from Clusters #2 and #4 obtained from Fig. 5. The different colored markers are used to represent the various morphological clusters. (**E**) Average Proportionality plots of the distribution of VSMC spheroids in each morphological cluster from the repeated random splitting of training and testing sets. Only testing sets were used. *p < 0.05, **p < 0.01, ***p < 0.001, and *ns* not significant. (**F**) Summary of the clustering analysis results.

The morphological features used in the previous analysis were focused more on differentiating circular and noncircular spheroids, which are limited to distinguishing fine-grained clusters among the noncircular spheroids in Clusters #2 and #4 (or Types 2 and 4). For a more precise analysis of VSMC spheroids treated with inhibitors of FAK (10 μM), Rac (20 μM), and Rho (20 μM), as shown in Clusters #2 and #4 (Fig. 5E), we performed further morphological clustering using a wide range of morphological features (Fig. 1C, referring to “noncircular spheroid clustering”). Fifteen features were selected to represent the morphologies of noncircular and dispersed/disrupted VSMC spheroids, including the *area*, *major axis length*, *minor axis length*, *eccentricity*, *solidity*, *extent*, *perimeter*, *convex area*, *circularity*, *aspect ratio*, *actual area*, *actual equivalent diameter*, *actual circularity*, and *number of holes*. The morphological features extracted from the spheroids from the dispersed/disrupted noncircular spheroids were plotted on a UMAP plot (Fig. 6A). Subsequently, clustering analysis with the community detection algorithm was performed, and a cluster number of four was determined by the local maximal silhouette value from the training set in the same manner as the initial clustering (Fig. 6B). The silhouette plot of the clustering outcome in the testing set (Fig. 6C) showed mostly positive values and very few negative values, indicating the presence of four distinct clusters. The four clusters of drug-treated spheroids with representative images were plotted on a UMAP plot (Fig. 6D). The spheroids treated with 20 μM Rac inhibitor were almost evenly spread out among the disrupted clusters, Types N1-4, while most of the VSMC spheroids treated with 10 μM FAK inhibitor were Types N1, N2, and N4, suggesting that Type N3 morphology arises when Rac activation is inhibited, but not when FAK phosphorylation is inhibited (Fig. 6E). Intriguingly, Type N3 was enriched (50%) when the VSMCs were treated with 20 μM Rho inhibitors, while Type N1 was depleted (5%). Overall, we identified spheroid morphological clusters of VSMCs with unique drug responses, suggesting the following: (i) Types N1 and N4 morphologies mostly depend on the disruption of FAK and Rac, (ii) Type N3 morphology mainly depends on the disruption of Rho and Rac, (iii) Type N2 morphology depends on the disruption of FAK, Rac, and Rho (Fig. 6E,F), and (iv) Cluster #3 morphology from the previous initial clustering analysis depends on the disruption of only Cdc42 (Figs. 5E, 6F). Regarding the robustness of the clustering, the data were assigned to the major clustering labels highly consistently as shown in Fig. S5C (Cluster #N1: 97.64%, Cluster #N2: 96.65%, Cluster #N3: 98.22%, Cluster #N4: 98.52%). Also, clustering of the entire dataset without the data splitting exhibited similar results (Fig. S6).

## Discussion

We used scaffold-free 3D spheroid culture systems to better mimic in vivo cellular microenvironments of complex tissues than traditional 2D monolayer cell culture by forming more complex cell-cell and cell-ECM interactions. Consequently, spheroids are considered valuable experimental models for investigating the effects of drugs on signaling pathways that control cellular functions^66–69^. Because a spheroid’s structure depends on the collective functions of its constituent cells (including their adhesion properties, stiffness, proliferation, apoptosis, and migration behaviors), spheroid morphology can be a very sensitive indicator of cellular responses to drugs or other manipulation. Recently, spheroid-based in vitro angiogenesis models have been explored to mimic in vivo vessel sprouting, with co-culture of endothelial cells and VSMCs or pericytes^70,71^. That model system recapitulates similar physiological architecture of healthy blood vessels and evaluates potential angiogenic modulators. In addition, Barnes et al. used VSMC spheroids to show that membrane type-1 matrix metalloproteinase expressed by VSMCs can limit the progression of proliferative atherosclerotic lesions in apoE-null mice^72^. Collectively, these recent studies suggested that 3D vascular spheroid systems can be used in vascular biology and pathology.

Atherosclerosis and vascular injury are characterized by neointimal hyperplasia and formation caused by VSMC accumulation and proliferation within the vessel wall^1,4,5,9–12^. In response to vascular injury, VSMCs transition from a highly differentiated state to a dedifferentiated state, in which they exhibit increased proliferation and constitute a substantial part of the neointima^1–3^. While this process resolves smaller injuries to the vessel wall, its persistence in chronic injury becomes pathological, generating cellular occlusions in the vessel lumen, reducing blood flow, and creating the need to resolve these changes. Thus, understanding how to control VSMC accumulation and proliferation would advance the effort to treat vascular diseases. Here, we created a 3D VSMC spheroid model to mimic VSMC-mediated neointima formation observed in the arterial wall, similar to in vivo animal models of restenosis, vascular injury, and atherosclerosis, and tested the effects of FAK, Rac, Rho, and Cdc42 inhibition on the formation and morphology of VSMC spheroids.

FAK and its downstream small GTPase proteins (Rac, Rho, and Cdc42) are reported to be key regulators of VSMC adhesion and proliferation, mostly by using a 2D cell culture system and several animal studies. Here, using a 3D VSMC spheroid model system, we showed that the inhibition of FAK phosphorylation led to marked disruption during the process of spheroid formation. This result suggests that FAK phosphorylation is required for VSMC spheroid formation potentially through N-cadherin-mediated cell-cell adhesion^12,73^, which is known to be regulated by Rac, Rho, and Cdc42^12,26,30,34^. Interestingly, we observed that inhibition of Rac and Rho activation with high dose drug treatment, but not Cdc42 inhibition, caused severe disruption of VSMC spheroid formation. Our data can be explained by previous observations from a study using stem cells showing that N-cadherin mediated cell-cell adhesion is modulated by Rac signaling^74^. Moreover, other previous studies demonstrated that there is an increase in Rac activity after a fine-wire vascular injury to the intimal layer, but VSMC-specific deletion of Rac and N-cadherin reduced neointima formation following vascular injury^12^. In addition, Feil et al. showed that N-cadherin-dependent cell-cell contacts destabilized when endogenous Rho was inhibited^75^. Collectively, these observations and our results suggest signaling pathways where FAK controls the process of VSMC spheroid formation through FAK-Rac- or FAK-Rho-mediated cell-cell adhesion.

The direct connection of Cdc42 to VSMC-mediated neointima formation in response to vascular injury or atherosclerosis remains largely unexplored. We showed that Cdc42 inhibition disrupted the formation of the spheroid but generated multiple small spheroids. This result might indicate that levels of N-cadherin expression are being rescued and functional N-cadherin-mediated cell-cell adhesion within each small spheroids restored. Cdc42 is known to regulate the attachment of actin fibers to the sites of cell-cell adhesion that are stabilized by the junctional proteins. Thus, inhibition of Cdc42 could have reduced levels of junction proteins other than N-cadherin such as occludin^76^, which might have resulted in disruption of the spheroids.

A salient result achieved by our HETEROID pipeline is that it computationally identifies the presence of heterogeneous morphologies (morphological variations) of the spheroids resulting from drug treatment with FAK, Rac, Rho, and Cdc42 inhibitors. We used VGG16-U-Net segmentation, which combines the U-Net and ImageNet-pretrained VGG16 models^47–50^. Unlike simple ML algorithms that use binary thresholding or filtering, deep learning is designed to learn more in-depth pixel information to identify spheroid edges (or boundaries). This design is particularly useful for brightfield or phase-contrast microscopy, which is more suitable for high-throughput applications than fluorescence microscopy. Indeed, our unsupervised ML algorithm combined with the clustering analysis approach identified that FAK, Rac, Rho, and Cdc42 inhibitors differentially affect the spheroid morphology of VSMCs. More specifically, we observed that FAK inhibition disrupted VSMC spheroid formation and morphology most likely through Rac but not Rho or Cdc42 (Fig. 6E). Furthermore, we identified that Cdc42 inhibition disrupted VSMC spheroid formation and morphology independent of the FAK-mediated signaling pathway (Figs. 5E, 6F). These new findings obtained by our HETEROID framework suggest that multiple distinct pathways govern the process of VSMC spheroid formation.

Zanoni et al. demonstrated that various morphologies of tumor spheroids exist that correspond to the viability of the tumor cells within the spheroid^77^. Moreover, a recent study on pancreatic cancer spheroids by Laurent et al. reported that the cells in multicellular tumor spheroids showed varying effects in response to antiproliferative drugs^78^. This variation in results was attributed to the size of the spheroids and the packing of cells in the spheroids. Thus, with our data obtained from VSMC spheroids with our computational framework, further investigation of global changes in transcriptome or proteome in VSMC spheroids using ‘Omics’ technologies will establish the molecular and functional significance of these distinct morphological clusters (or types) and result in understanding the morphology of the biological relationship. These further investigations will lead us to (i) determine the biological and functional roles of FAK, Rac, Rho, and Cdc42 during the process of VSMC spheroid formation and (ii) understand why only a subpopulation of VSMCs underlying the site of vascular injury is actively involved in hyperplasia during the process of VSMC-mediated neointima formation^6^. Therefore, these previous studies and our current and future studies will potentially be a development in helping to identify specific drug targets for VSMC spheroids based on heterogeneous responses in spheroid morphologies.

## Conclusion

This study tested the effect of FAK and its downstream small GTPase manipulation on VSMC spheroid formation and used a machine learning approach to reveal heterogeneous responses to FAK, Rac, Rho, and Cdc42 inhibition on VSMC spheroid morphology. The machine learning framework was designed to extract morphological features of the spheroids from phase-contrast images without using fluorescent staining. The results shown in our study suggest that there is significant drug-induced heterogeneity in spheroid formation and morphology, which has been overlooked in previous analyses and studies. These detected subpopulations (clusters or types) could give rise to the biological significance of various drug responses upon further study. For instance, examining the effects of drug treatment on global changes in the transcriptome or proteome and on viability, proliferation, or apoptosis in VSMC spheroids within each morphological cluster (shown in Figs. 5D and 6D) can further reveal the biological and functional significance associated with spheroid morphological changes. The biological and functional significance of FAK-, Rac-, Rho-, and Cdc42-mediated spheroid formation and morphology will be a stepping stone in identifying potential pharmaceutical drug targets for preventing neointima formation or hyperplasia. This is our first step towards developing an automated method for quickly and precisely analyzing the heterogeneity of VSMC spheroids using machine learning. This machine learning approach can be used to study the effects of different pharmacological drugs on VSMC spheroid models for better characterization of the pathophysiologic progression of diseases such as atherosclerosis, hypertension, and restenosis. The long-term goal of future studies is to be able to use machine learning algorithms to assist in personalized medicine by testing the effects of pharmacological drugs on patient-specific spheroids before prescribing drugs to patients.

## Methods

### Cell culture and drug treatment

Primary human vascular smooth muscle cells (Catalog No.: 354-05a; Lot No.: 2435; Source: normal human aorta from 33-year-old male; Cell Applications) and primary mouse VSMCs (Catalog No.: C57-6080; Lot No.: 090915; Source: normal mouse aorta from mixed-gender mice; Cell Biologics) were cultured as previously described^8,11,79^. The human and mouse VSMCs were used at passages 3–8 and 3–5, respectively. For serum starvation to synchronize cells, near-confluent VSMCs were incubated for 48 h in serum-free DMEM containing 1 mg/ml heat-inactivated, fatty-acid free bovine serum albumin (BSA; Tocris). For the FAK, Rac, Rho, and Cdc42 pharmacologic inhibitor experiments, cells in suspension culture were treated with 10 μM FAK-specific inhibitor PF573228^11^ (Sigma), 5–20 μM Rac-specific inhibitor EHT1864^58^ (Tocris), 5–20 μM RhoA-specific inhibitor Rhosin^60^ (Tocris), or 5–10 μM Cdc42-specific inhibitor ML 141^63^ (Tocris) reconstituted in dimethyl sulfoxide (DMSO) for selected times up to 24 h.

### Generation of VSMC spheroids

VSMC spheroids were generated by the hanging drop technique^37,38^. Briefly, serum-starved cells were trypsinized, centrifuged, and resuspended in fresh high-glucose (4.5 g/L) DMEM containing 10% fetal bovine serum (FBS). The cell suspension was diluted to approximately 2000 cells per 20 μL of high-glucose DMEM with 10% FBS and treated with either pharmacological inhibitors (PF573228, EHT1864, Rhosin or ML141) or DMSO (vehicle control) before plating. Approximately 20–30 cell suspension droplets were dispensed on the hydrophobic surface of the lid of a 100-mm tissue culture dish. Note that the drops were seeded sufficiently apart to avoid the mixture of each adjacent drop. To prevent the evaporation of the drops, a water reservoir (hydration chamber) was made by adding 8 ml of sterile water and 2 ml of DMEM at the bottom of the tissue culture dish. The lid was then carefully inverted so the cells remained suspended and were allowed to aggregate by gravity. The cultures were maintained at 37 °C and 10% (human VSMCs) or 5% (mouse VSMCs) CO_2_. VSMC spheroids formed after 24 h of incubation. Images were taken at 10X magnification using an upright microscope (Olympus BH-2) equipped with a digital camera (AmScope) to monitor spheroid formation.

### Protein extraction and immunoblotting

Total cell lysates were prepared from human and mouse VSMC spheroids. The spheroids were collected in 1.7 ml Eppendorf tubes, centrifuged, and extracted in 1× TNE lysis buffer [50 mM Tris–HCl (pH 8.0), 250 mM NaCl, 2 mM EDTA, 1% Nonidet P-40, plus a protease and phosphatase inhibitor cocktail composed of a proprietary mix of AEBSF, aprotinin, bestatin, E64, leupeptin, and pepstatin A to promote broad-spectrum protection against endogenous proteases]. Equal amounts of extracted protein were mixed with 5× sample buffer [250 mM Tris–HCl (pH 6.8), 10% SDS, 50% glycerol, 0.02% bromophenol blue, 10 mM 2-mercaptoethanol], denatured by boiling samples at 100 °C, subjected to 8% sodium dodecyl sulfate (SDS) polyacrylamide gel electrophoresis and transferred to a polyvinylidene difluoride (PVDF) membrane. The PVDF membranes were blocked with either 5% BSA or 5% milk in 1× TBST (Tris-buffered saline, 0.1% Tween 20) for 1 h and probed with primary antibodies against phospho-FAK (Invitrogen, 44-624G; diluted 1:500) and GAPDH (Proteintech, 60,004-I-Ig; diluted 1:5000). After overnight incubation at 4 °C, the PVDF membranes were washed with 1× TBST and probed with horseradish peroxidase-conjugated secondary antibodies (Bio-Rad) for 1–2 h at room temperature. Immunoblot signals were detected using chemiluminescence (Bio-Rad).

### Deep learning-based segmentation of the spheroid images

i. *Data labeling* A deep neural network was trained to automatically segment the spheroid images. To train the deep neural network, we selected a set of 23 diverse VSMC spheroid images from the whole dataset. Depending upon the drug treatment conditions, the contrast of the spheroid edges was highly varied. Therefore, we selected these training images to represent these variations of edge contrast. And, then spheroid boundaries were manually drawn using the Pixel Annotation Tool (https://github.com/abreheret/PixelAnnotationTool). The images of the hand-drawn boundaries were used to generate binary mask images. The masks were used as the training dataset from which the deep neural network learned to identify the boundaries of the spheroids.
ii. *Training dataset preparation* We randomly cropped 200 image patches (128 × 128 pixels) from each raw image (1128X832 pixels). In total, we had 4600 (200 × 23) image patches for the training. 60% of the cropped patches contained the edge boundary, and 40% contained only the foreground or background (not containing edges). After the image patches were cropped, the means and standard deviations of the collected image patches were calculated for further normalization. After preprocessing, we split them into training and validation sets at a ratio of 8:2 (3200 for training and 800 for validation). Then, the standard data augmentation process was applied using the ImageDataGenerator class implemented in the Keras package. The parameters used in ImageDataGenerator for data augmentation are as follows: rotation_range = 90; horitontal_flip = true; vertical_flip = true fill_mode = ‘reflect’.
iii. *Training the deep neural network* The deep neural network is divided into two parts: an encoder that extracts image features and a decoder that identifies the edge locations using the extracted features. The VGG16 model was used in the encoder and extracted features at multiple levels. The initial weights for the VGG16 model were from the pretrained model using the ImageNet DB. The VGG16 weights were fixed during training. The decoder from U-Net was incorporated to combine the VGG16 features to generate the segmented images. The generated masks were then overlapped with raw images to visualize the learning efficiency of the algorithm. The binary cross-entropy loss was used as a loss function for training. Adam was used as the optimizer, the initial learning rate was set to 5 × 10^–4^, and the other parameters were set to the default values in Keras. To avoid overfitting, we used an early stopping strategy. We stopped the training when the validation loss did not decrease by 0.0001 in three consecutive epochs. The maximum number of epochs was 30, and the batch size was 32. Neural network training was performed using Keras with the TensorFlow backend on a computer equipped with an NVIDIA GTX 1080 Ti or Titan X GPU.
iv. *Performance evaluation of edge localization* We used the labeled data that were not included in the training process (training/validation sets) for the performance evaluation. We generated binarized mask images by thresholding the softmax output images using a threshold value of 50 (> 50: foreground, ≤ 50: background. Pixel value range: 0–255). The edge images were predicted for all the image frames, including the labeled and unlabeled images. We used the predictions of labeled images not used for training to evaluate the edge localization performance.
  We calculated Dice coefficients to evaluate the segmentation performance along the edge boundary as follows. We applied a binary AND operation between the dilated edge masks and the ground-truth masks and the predicted masks from VGG16-U-Net using the *bitwise_and* function in the OpenCV package. Then, we calculate the Dice coefficient between the ground truth and the predictions for each image frame using the following formula:
  $$Dice\left(A,B\right)= \frac{2\left|A\cap B\right|}{\left|A\right|+\left|B\right|}$$
  where $\left|A\cap B\right|$ are the common elements between sets $A$ and $B$ and $\left|A\right|$ and $\left|B\right|$ are the numbers of elements in sets $A$ and $B$.
v. *Comparison with the baseline models* We compared our model, VGG16-U-Net with the baseline models, U-Net and VGG16-U-Net-NP (a non-pretrained version of VGG16-U-Net). The weights of U-Net and VGG16-U-Net-NP were randomly initialized. We repeated five-fold cross-validation 10 times. For each cross-validation, a single subsample was used for testing and the other four subsamples were used for training. Then, we performed the statistical analyses using the Dice coefficients from the cross-validations.

### Unsupervised spheroid morphology learning

We used all 856 images of the spheroids treated with DMSO and the inhibitors of FAK, Rac, Rho, and Cdc42 as described in Table 1 (the training images for deep learning segmentation were excluded from these images).

i. *Feature extraction* The segmented spheroid images were processed for extracting the morphological features. The morphological features were used during unsupervised learning to characterize the responses of the spheroids to drug treatments. The morphological features extracted are listed in the following clustering analysis section. Once the features were quantified, feature standardization was used to achieve a zero mean and unit variance in each experiment to remove batch effects, followed by principal component analysis in each step of clustering below. The feature vectors extracted from the spheroids were visualized by a UMAP plot.
ii. *Clustering analysis* The feature vectors generated from the spheroid images were analyzed to identify the presence of morphological subpopulations. The community detection clustering analysis was performed using the feature vectors in two steps: the clustering of whole spheroids (step 1) and the clustering of noncircular and disrupted spheroids (step 2). The number of clusters was determined by the maximum of silhouette values in the clustering results^80^.

**Table 1:** The numbers of images used for unsupervised spheroid morphology learning.

Inhibition	DMSO	FAK	Cdc42		Rac			Rho			Total
Dose (μM)	–	10	5	10	5	10	20	5	10	20	
# of images	109	112	87	88	93	44	85	87	88	63	856

We set up a two-step clustering pipeline based on our observation about the morphological changes by the drug treatments. The control (DMSO) spheroids are mostly circular, and the roundness of the spheroids was affected dramatically and globally by the drug treatments. The control or mildly affected spheroid are well described by the morphological features related to the roundness of the spheroids while highly irregular spheroids require more comprehensive features. Therefore, in the first step, we used the limited features set related to the roundness. After we identify noncircular and disrupted spheroids, we use more morphological features to further cluster them.

- *Clustering of whole spheroids (step 1)* Five morphological features relating to the roundness of the spheroids were used to cluster the spheroids. The features were the *circularity* (4π area/perimeter^2^), *extent* (the spheroid colony with the largest area), *solidity* (proportion of the pixels in the convex polygon that is also in the region), *eccentricity* (a measure of how much an ellipse is different from being circular. An ellipse whose eccentricity is 0 is a circle), and the *number of colonies*. Then, PCA was applied to these roundness-related features. The resulting clusters were expected to show clusters of spheroid images with round morphologies and clusters with noncircular and disrupted morphologies.
- *Clustering of noncircular and disrupted spheroids (step 2)* Clusters containing the noncircular spheroids (Clusters #2 and #4; Fig. 5E) were selected, and more features were extracted from the selected spheroids for further clustering analysis. Additional morphological features were extracted from the noncircular spheroid clusters. These morphological features were more focused on exploring morphology with various complex patterns of the spheroid. The morphological features extracted include the *area* (the area of all the pixels in the largest external contour), *major axis length* (the length in pixels of the major axis of the ellipse that has same normalized second central moments as the region), *minor axis length* (the length in pixels of the minor axis of the ellipse that has same normalized second central moments as the region), *eccentricity*, *solidity*, *extent* (the ratio of the number of pixels in the region to the number of pixels in total bounding box, returned as a scalar), *perimeter* (distance around the boundary of the region, returned as a scalar), *convex area* (number of pixels in the bounding box of the region), *circularity*, *aspect ratio*, *actual area*, *actual equivalent diameter* (the diameter of a sphere with the same volume as the region), *actual circularity*, and *number of holes* (number of internal contours). Then, PCA was applied to these morphological features.
- *Determination of the number of clusters* We randomly split the data into training and testing sets in equal proportion. By varying the number of nearest samples in the community detection algorithm, we generated the clustering results for different numbers of clusters in the training set. We calculated the silhouette values of the clustering results of the different number of clusters. We determined the optimal number of clusters from the local maximum silhouette values. Then, we performed the same clustering analysis for the testing set using that number of clusters.
- *Clustering evaluation* We randomly split the data into training and testing sets in equal distribution 100 times. We performed the clustering in each splitting and evaluated the consistency of the clustering results. Because the sequences of the cluster labels in each run of clustering are random, we visually checked the clustering result in each run and manually reassigned the labels to the same types of clusters. We counted the cluster label assignments of individual spheroid images from the repeated clustering, and the majority cluster label for each image was determined. Then we calculated how often the majority labels correspond to the cluster labels in each run of clustering. The consistency of clustering is indicated by how close the cluster assignments to the majority labels are to 100%.
- *Drug effects* We quantified the drug effect based on the cluster proportion calculation as follows. We counted the number of spheroid images of each cluster for the control and drug treatment experiments. Then, the number was resampled using the bootstrap() function in MATLAB to build 10,000 different bootstrapped datasets, and the distribution of the proportions in each experiment was generated. Based on these distributions, p-values were calculated by estimating the probability that the cluster proportion was greater than or less than that of the other experiment. In addition, the 95% confidence intervals of the proportions were estimated by bootci() function in MATLAB.

### Statistical analysis

The data are presented as means ± standard errors of the mean (SEM) for the indicated number of independent experiments. The data in Figs. 2B and S1B were analyzed using Student’s two-sided paired t-tests. The Kolmogorov–Smirnov normality test was performed and the null hypothesis of the normality could not be rejected. The statistical analysis in Fig. 4D was performed by Wilcoxon rank sum test, which does not rely on the normality assumption. The statistical analysis in Fig. 6E was performed by bootstrap sampling where the cluster labels were resampled to build 10,000 bootstrapped dataset and p-values were calculated by estimating the probability that the proportion of one cluster is different from that of the other. The differences with p-values less than 0.05 were considered statistically significant.

## Supplementary Information

Supplementary Information.

## Data Availability

The datasets used in the current study are available from the corresponding authors on reasonable request.

## Code availability

The code used in the current study is available from the corresponding authors upon reasonable request.
