# Supplementary material for: A machine learning pipeline revealing heterogeneous responses to drug perturbations on vascular smooth muscle cell spheroid morphology and formation
Source: Sci Rep. 2021 Dec 2;11:23285. doi: 10.1038/s41598-021-02683-4 (PMC8640073; doi:10.1038/s41598-021-02683-4)
Supplement: Supplementary file 1 — Supplementary Information. [file 41598_2021_2683_MOESM1_ESM.pdf]

Supplementary data

A machine learning pipeline revealing heterogeneous responses to drug perturbations on vascular smooth muscle cell spheroid morphology and formation

Kalyanaraman Vaidyanathan[1#], Chuangqi Wang[2#], Amanda Krajnik[1], Yudong Yu[2], Moses Choi[2], Bolun Lin[3], Junbong Jang[4], Su-Jin Heo[5], John Kolega[1], Kwonmoo Lee[2,4,6*], Yongho Bae[1*]

[1]Department of Pathology and Anatomical Sciences, Jacobs School of Medicine and Biomedical Sciences, University at Buffalo, State University of New York, Buffalo, NY 14203, USA. [2]Department of Biomedical Engineering, Worcester Polytechnic Institute, Worcester, MA 01609, USA. [3]Department of Computer Science, Worcester Polytechnic Institute, Worcester, MA 01609, USA. [4]Vascular Biology Program, Boston Children's Hospital, MA 02115, USA. [5]Department of Orthopedic Surgery, Perelman School of Medicine, University of Pennsylvania, Philadelphia, PA 19104, USA. [6]Department of Surgery, Harvard Medical School, Boston, MA 02115, USA

[#]These authors contributed equally: Kalyanaraman Vaidyanathan and Chuangqi Wang.

*Correspondence: yonghoba@buffalo.edu and kwonmoo.lee@childrens.harvard.edu

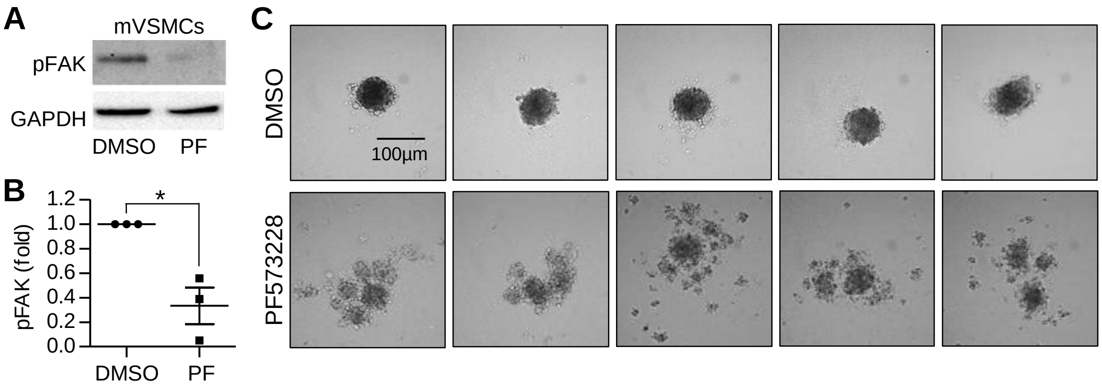

Figure S1: FAK inhibition disrupts mouse VSMC spheroid formation. Mouse VSMC (mVSMC) spheroids were made in the presence of either DMSO (vehicle control) or 10 µM PF573228 (PF) in high glucose DMEM containing 10% serum. Total cell lysates were immunoblotted (A) for phosphorylated FAK at Tyr397 (pFAK) and GAPDH. The bar graph displays pFAK levels normalized to DMSO control (B). (C) Cultures were imaged after 24 hours of incubation using an upright microscope. n=3 (A-B) and n=12 (C) biological replicates were used. $^*p<0.05$.

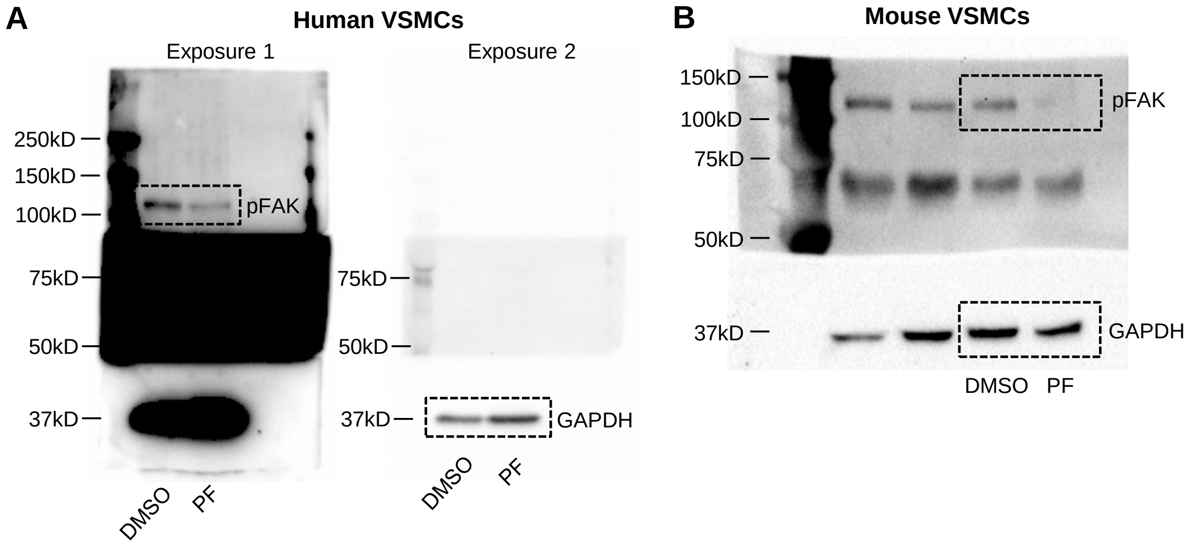

Figure S2: Uncropped western blot images presented in the main (A, human VSMCs) and supplementary (B, mouse VSMCs) figures. Molecular weight markers are indicated.

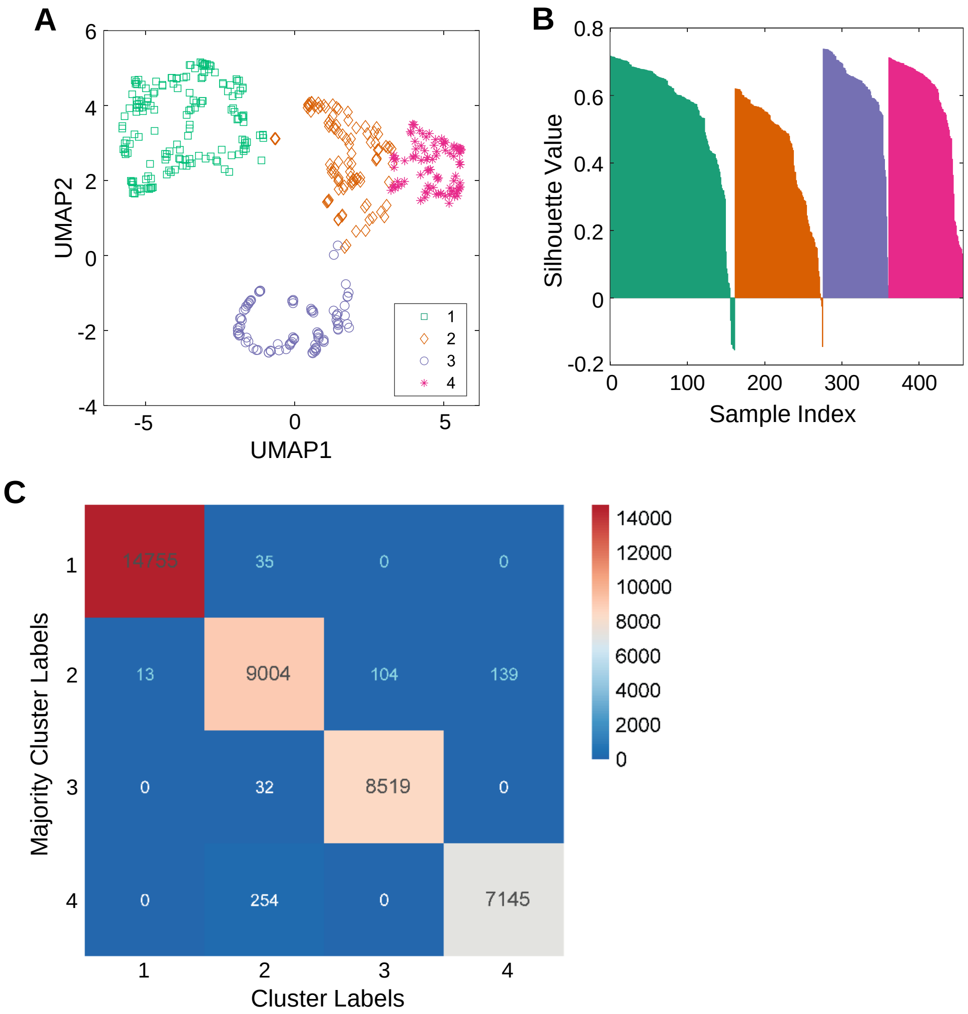

Figure S3: Initial clustering analysis in response to inhibitors of FAK, Rac, Rho, and Cdc42 using the training set and the robustness evaluation of the clustering using the testing set. (A) Four morphological features extracted from human VSMC spheroid images are displayed on a UMAP plot (only samples in the training set). (B) Silhouette plots for the clustering results on the training set. (C) The consistency matrix of the clustering results using the testing set.

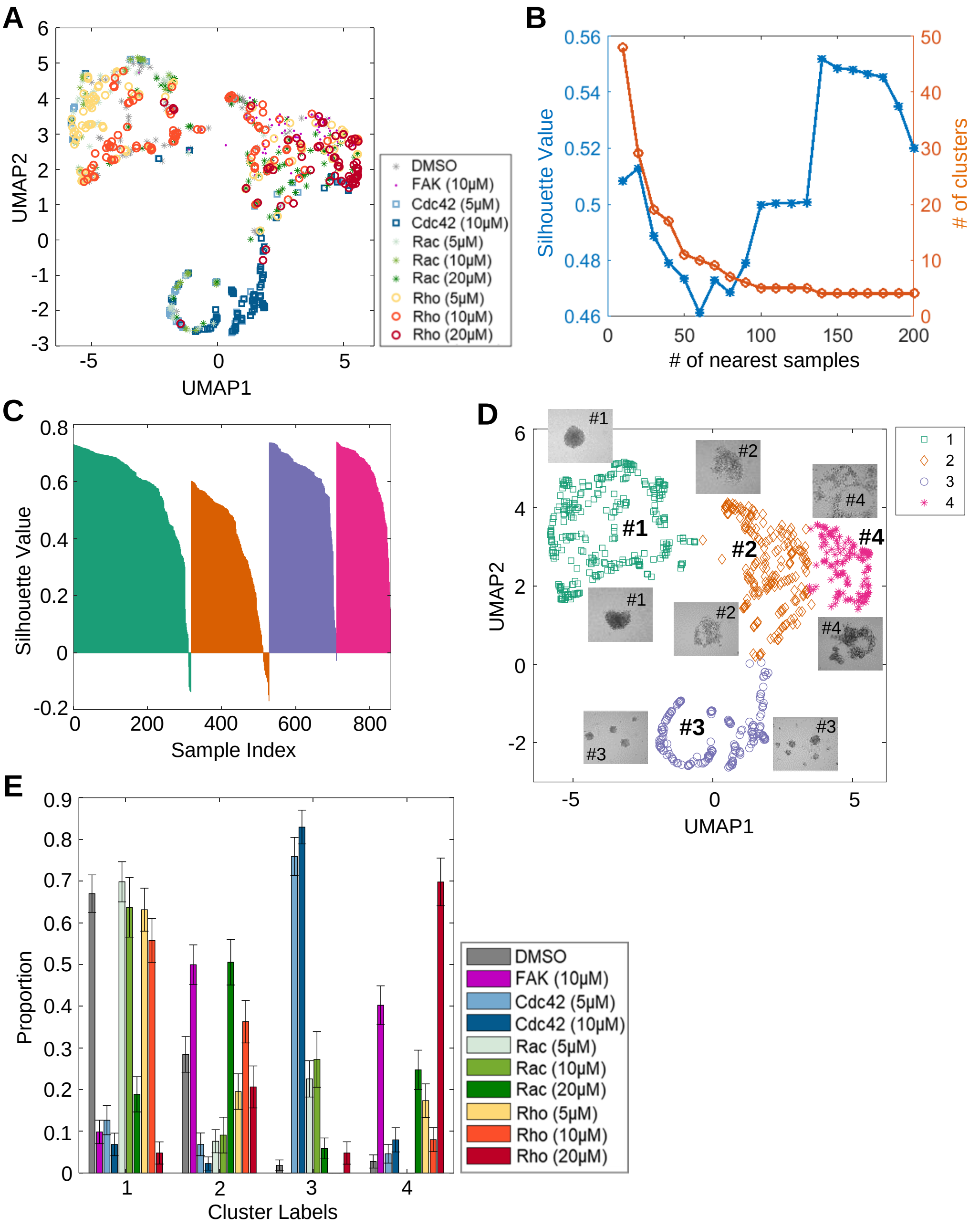

Figure S4: Initial morphological clustering analysis identifying distinct clusters in response to inhibitors of FAK, Rac, Rho, and Cdc42 in the entire dataset. (A) Four morphological features extracted from human VSMC spheroid images are displayed on a UMAP plot. The different colored markers are used to represent the various drug treatments: FAK (PF573228), Rac (EHT1864), Rho (Rhosin), and Cdc42 (ML141). (B) The number of nearest samples was evaluated, and the silhouette values were plotted. A cluster number of four was selected. (C) Silhouette plots for the clustering results. (D) Four morphological clusters plotted on the UMAP plot were observed based on the "roundness of the spheroid". The different colored markers are used to represent the various morphological clusters with the drug treatments. The most representative images were overlayed on each cluster (2 images per cluster) in the UMAP plot. Clusters #1 and #3 showed spheroids with circular morphologies, and the spheroids in Clusters #2 and #4 showed noncircular and dispersed/disrupted morphologies. Only the samples in the test dataset were shown. (E) Average proportionality plot of the distribution of VSMC spheroids in each morphological cluster with the drug treatments.

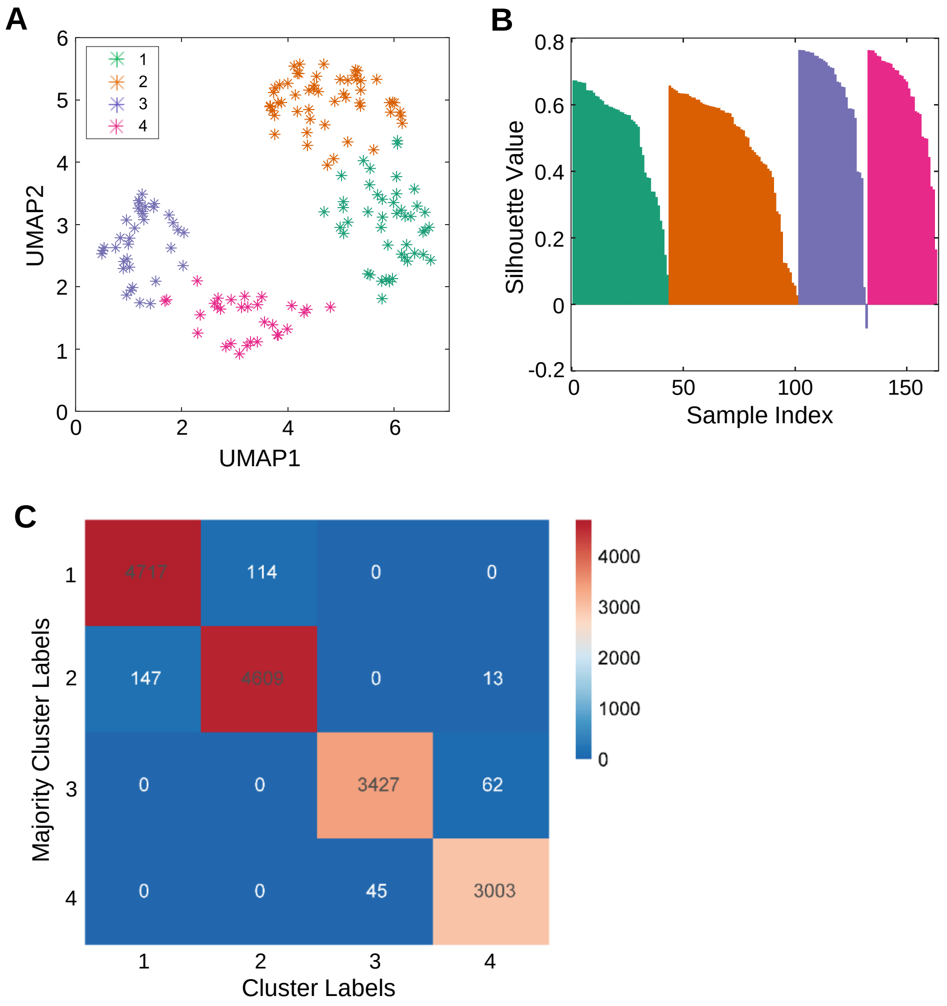

Figure S5: Clustering analysis of noncircular spheroids and the presence of distinct clusters with disrupted morphologies using the training set and the robustness evaluation of the clustering using the testing set. (A) Four morphological features extracted from human VSMC spheroid images are displayed on a UMAP plot (only samples in the training set). (B) Silhouette plots for the clustering results on the training set. (C) The consistency matrix of the clustering results using the testing set.

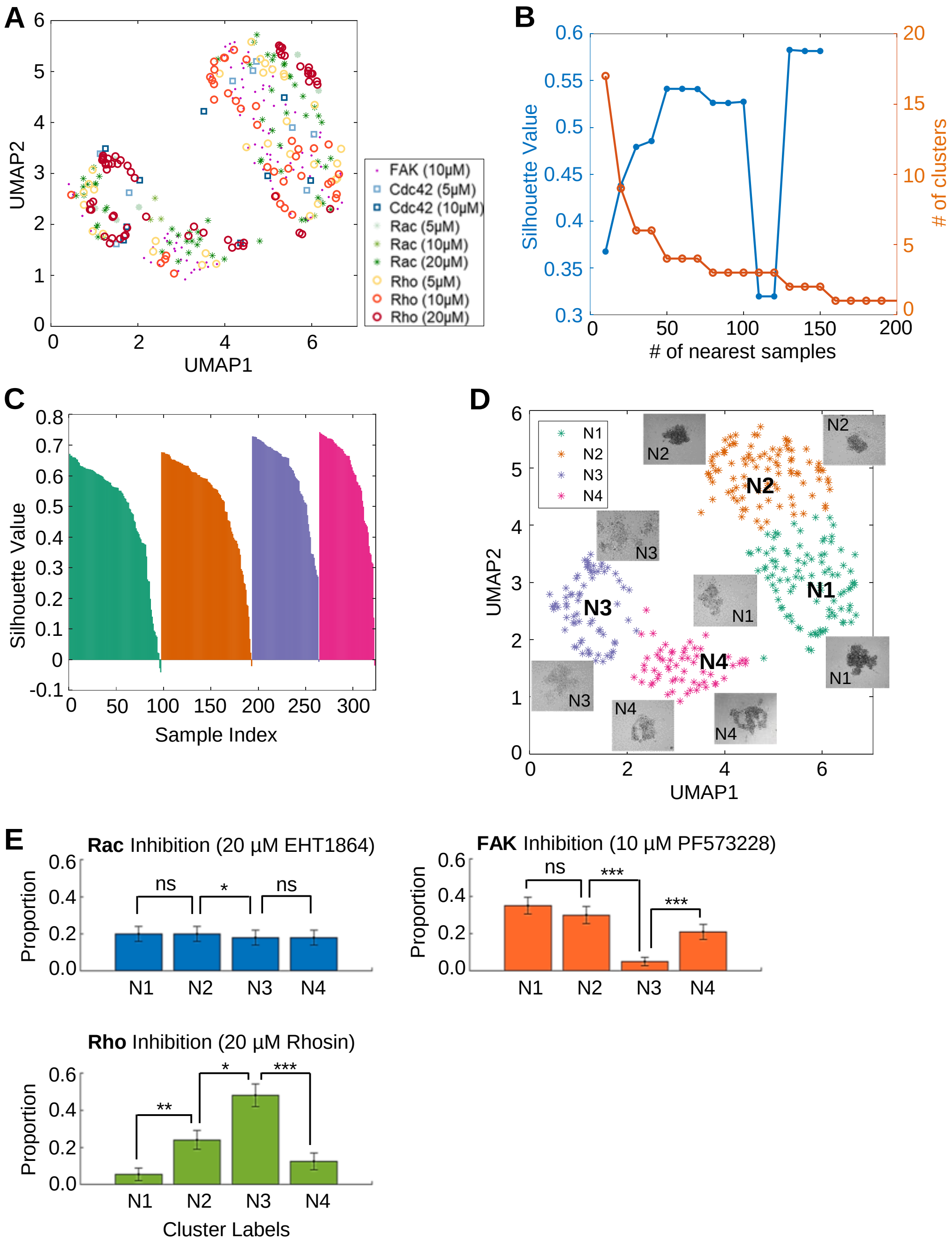

Figure S6: Clustering analysis of noncircular spheroids and the presence of distinct clusters with disrupted morphologies in the entire dataset. (A) Fifteen morphological features extracted from human VSMC spheroid images from Clusters #2 and #4 were visualized on UMAP plots. The different colored markers are used to represent the various drug treatments. (B) The number of nearest samples was evaluated, and the silhouette values were plotted. A cluster number of 4 was selected. (C) Silhouette plots for the clustering results. (D) Four morphological clusters were observed in the test set based on the noncircular spheroids from Clusters #2 and #4 obtained from Fig. 5. The different colored markers are used to represent the various morphological clusters. (E) Average Proportionality plots of the distribution of VSMC spheroids in each morphological cluster. *p<0.05, **p<0.01, ***p<0.001, and ns: not significant.
